# A High-Density SNP Map for Accurate Mapping of Seed Fibre QTL in *Brassica napus* L

**DOI:** 10.1371/journal.pone.0083052

**Published:** 2013-12-26

**Authors:** Liezhao Liu, Cunmin Qu, Benjamin Wittkop, Bin Yi, Yang Xiao, Yajun He, Rod J. Snowdon, Jiana Li

**Affiliations:** 1 College of Agronomy and Biotechnology, Southwest University, Beibei, Chongqing, China; 2 Department of Plant Breeding, Interdisciplinary Research Centre for Biosystems, Land Use and Nutrition, Justus Liebig University, Giessen, Germany; 3 National Key Laboratory of Crop Genetic Improvement, National Subcenter of Rapeseed Improvement in Wuhan, Huazhong Agricultural University, Wuhan, China; Nanjing Forestry University, China

## Abstract

A high density genetic linkage map for the complex allotetraploid crop species *Brassica napus* (oilseed rape) was constructed in a late-generation recombinant inbred line (RIL) population, using genome-wide single nucleotide polymorphism (SNP) markers assayed by the *Brassica* 60 K Infinium BeadChip Array. The linkage map contains 9164 SNP markers covering 1832.9 cM. 1232 bins account for 7648 of the markers. A subset of 2795 SNP markers, with an average distance of 0.66 cM between adjacent markers, was applied for QTL mapping of seed colour and the cell wall fiber components acid detergent lignin (ADL), cellulose and hemicellulose. After phenotypic analyses across four different environments a total of 11 QTL were detected for seed colour and fiber traits. The high-density map considerably improved QTL resolution compared to the previous low-density maps. A previously identified major QTL with very high effects on seed colour and ADL was pinpointed to a narrow genome interval on chromosome A09, while a minor QTL explaining 8.1% to 14.1% of variation for ADL was detected on chromosome C05. Five and three QTL accounting for 4.7% to 21.9% and 7.3% to 16.9% of the phenotypic variation for cellulose and hemicellulose, respectively, were also detected. To our knowledge this is the first description of QTL for seed cellulose and hemicellulose in *B. napus*, representing interesting new targets for improving oil content. The high density SNP genetic map enables navigation from interesting *B. napus* QTL to *Brassica* genome sequences, giving useful new information for understanding the genetics of key seed quality traits in rapeseed.

## Introduction

Precise linkage map construction is the first step for mapping of quantitative trait loci (QTL) and comparative genome analysis of interesting QTL regions. In oilseed rape (*Brassica napus* L.) a large number of low-density genetic maps, generated using electrophoretic marker systems like restriction fragment length polymorphism (RFLP), amplified fragment length polymorphism (AFLP) and simple sequence repeat (SSR), have been used to map qualitative and quantitative trait loci for a large number of traits [1]–[9].

A major disadvantage of many of these previous *B. napus* QTL mapping studies was an inability to derive and compare exact chromosomal locations for regions of interest. This situation can be improved using sequence data from tightly-linked markers, particularly as genome sequences become available for *Brassica* crops [10]. It will be extremely useful for closer examination of QTL and potential positional gene cloning to be able to navigate directly form genetic map positions to the genome sequence. In genetic maps with low marker densities, or higher-density maps based on anonymous markers like AFLP or SRAP, this is possible only by labour-intensive development and addition of sequence-based markers to saturate regions of interest.

The most abundant and simple DNA markers for mapping and other applications are single nucleotide polymorphisms (SNPs). Today SNPs have become the marker of choice in most species for genome-wide association studies (GWAS), phylogenetic analyses, marker-assisted selection, bulked segregant analysis and genomic selection. In 2012 an international *Brassica* SNP consortium produced a 60,000 (60 k) SNP Infinium genotyping array for *B. napus*, in cooperation with Illumina Inc., San Diego, CA, USA [11]–[12]. This introduced a very low-cost and efficient method for high-density, sequence-based, genome-wide polymorphism screening in *B. napus* populations.

The use of high density genetic maps can greatly improve the precision of QTL localisation and the accuracy of effect estimates for detected QTL, especially for small and medium sized QTL [13]. Especially with the development of automated sequencing and genotyping technologies, many high density linkage maps have been constructed in different crops including oilseed rape/canola (*B. napus*). For example, Sun et al. [14] constructed an ultradense genetic map consisting of 13351 SRAP markers covering 1604.8 cM in *B. napus*. Raman et al. (2012a) [15] used diversity array technology (DArT) markers to construct a linkage map for a doubled haploid (DH) mapping population of *B. napus*, and further constructed a consensus linkage map for genetic dissection of qualitative and quantitative traits (Raman et al. 2012b) [16]. The polymorphisms in the high throughput DArT marker system derive from restriction enzyme recognition sites and insertion/deletions (InDels). On the other hand, large fixed panels of robust SNP markers on widely-used array platforms are highly reproducible in different genetic backgrounds. A major advantage of genetic maps based on (public) genome-wide SNP screening arrays is the high occurrence of consensus markers for integration and alignment of maps and QTL, both to each other and also to reference genome sequences. Delourme et al. [17] developed an integrated *B. napus* map comprising 5764 SNP and 1603 PCR markers covering a total genetic length of 2250 cM. Chen et al. [18] also constructed a high density *B. napus* bin map, using a modified double-digested restriction-associated DNA sequencing (ddRADseq) approach.

Rapeseed is grown worldwide for vegetable oil and biodiesel production, and after oil extraction it also provides a high quality meal used primarily for livestock feeding [19]. Yellow-seeded *B. napus* is considered advantageous for the meal quality due to a thinner seed coat and higher protein content [20] along with reduced quantities of non-energetic fibre (cellulose and hemicellulose) and anti-nutritional polyphenolics (acid detergent lignin: ADL) [21]. Undigestible fiber, a major antinutritional component in rapeseed meal, can be reduced by breeding of light-seeded cultivars, whereas non-energetic cellulose and hemicellulose share photosynthesis products with seed oil and protein and are therefore important pleiotropic contributors to the agronomic value of the seed.

Numerous genetic mapping studies of seed colour loci have been reported in *Brassica* species using different biparental populations and marker technologies [22]–[35]. Many of these studies revealed QTL with different effects in different genetic backgrounds. A number of studies in *B. napus* suggested that one major locus on chromosome A09 explained most of the phenotype variation for both seed colour and meal quality traits in the most important *Brassica* oilseed crop [36]–[40]. However, attempts to saturate this QTL with markers [38], [41] have revealed possible chromosome rearrangements that make it difficult to find markers and genes with close physical linkage to the QTL. Alignment of QTL from all of these different studies has also been rendered difficult by this complication [41], and because of a lack of consensus markers spanning the QTL in the different studies. Whereas most PCR-based markers are polynary markers, especially in the allopolyploid *B.napus*, the Illumina type II beadtype SNPs utilized in the present study are robust binary markers which can be compared directly among diverse genetic backgrounds.

In this study we constructed a new, high density genetic map of *B. napus*, using a homozygous, F_9_ RIL population genotyped with the *Brassica* 60 k Infinium SNP array. To our knowledge this is the first high-density *B. napus* genetic map to be published with genotype data from the new *Brassica* 60 k SNP array. The map was used to precisely map QTL for seed colour, anti-nutritional seed ADL content, non-energetic seed cellulose and hemicellulose content using trait data from four field environments. The results provide tightly-linked and physically adjacent markers for breeding of these important agronomic traits in rapeseed, and a means to navigate directly from interesting map intervals to *Brassica* genome sequences as a key step in map-based QTL cloning.

## Materials and Methods

### Mapping population and trait analysis

A population of 172 F_9_ RILs was derived by single seed descent from F_2_ offspring of a cross between the Chinese semi-winter oilseed rape parental lines GH06 (yellow seeds, low seed fibre content) and P174 (black seeds, high seed fibre content). Seed quality traits were measured on self-pollinated seed samples collected from field evaluations of the RIL population in four different environments over two years as follows: 2008 at the agricultural field station of Justus Liebig University Giessen at Weilburger Grenze (central Germany), in the breeding nursery of the German plant breeding company NPZ Lembke KG in Hohenlieth (northern Germany) and in the breeding nursery of Southwest University in Beibei, Chongqing (southwest China), and 2009 again at the field station Weilburger Grenze in Giessen. No specific permissions are required to grow conventional oilseed rape on these agricultural locations. Inflorescences of up to three randomly-chosen plants in each plot were covered in pollen-proof bags at the onset of flowering to prevent cross-pollination. Self-pollinated seeds were collected in the bags at maturity for quality analysis.

All trials were performed with a plot size of 4.5 m^2^ (1.5 m×3 m) with 5 or 6 rows per plot depending on the location. Measurements for seed colour and fibre components on the self-pollinated seeds were obtained by near-infrared reflectance spectroscopy (NIRS) using an NIR System 6500 with WinISI II software (FOSS GmbH, Rellingen, Germany). Phenotype values for seed colour (visual light absorbance), acid detergent lignin (ADL; % seed dry weight), acid detergent fibre (ADF; % seed dry weight) and neutral detergent fibre (NDF; % seed dry weight) were extrapolated from NIRS spectra using the NIRS calibrations for these traits as described by Wittkop et al. [20]. NIRS-derived estimates for each trait and genotype were averaged over two technical repetitions. In the RIL population, mean trait values were averaged from up to three self-pollinated plants from each genotype over the four environments. The predicted means (eblups) were used for QTL identification of the seed color and fiber traits. Cellulose concentrations were calculated as the difference between NDF and ADF, hemicellulose as the difference between ADF and ADL. Basic statistical analysis of the phenotype data was performed using Microsoft Excel 2010.

### SNP marker analysis

The Brassica 60 K SNP BeadChip Array was used to genotype 172 RIL lines and parental lines. This array, which successfully assays 52,157 Infinium Type II SNP loci in *B. napus*, was developed by an international consortium using preferentially single-locus SNPs contributed from genomic and transcriptomic sequencing in genetically diverse *Brassica* germplasm (Isobel Parkin, Agriculture and AgriFood Canada, unpublished data).

Total genomic DNA was extracted from 150 mg of leaf tissue from 5 young seedings per RIL and from the two parental lines, using DP321-03 DNA extraction kits (Tiangen, Beijing, China). The DNA samples were diluted to 50 ng/μL. The SNP analysis was done in National Key Laboratory of Crop Genetic Improvement, National Subcenter of Rapeseed Improvement in Wuhan, Huazhong Agricultural University, 430070 Wuhan, China. DNA sample preparation, hybridisation to the BeadChip, washing, primer extension and staining were performed according to the work flow described in the Infinium HD Assay Ultra manual. Imaging of the arrays was performed using an Illumina HiSCAN scanner after BeadChip washing and coating. Allele calling for each locus was performed using the GenomeStudio genotyping software v2011 (Illumina, Inc.). Cluster definition was based on genotype data from 216 *B. napus* lines (9 Beadchips) including the 172 RILs and parental lines from this population and 42 additional *B. napus* genotypes. SNP markers were named using SNP plus index numbers assigned by GenomeStudio, followed by the chromosome number. Positions of A-genome SNPs were provided by the array manufacturer, while C genome SNP source sequences were subjected to a BLAST search against the *B.oleracea* genome database (BRAD, http://brassicadb.org/) to locate chromosome positions (E value < = 1e-50).

### Linkage analysis and QTL mapping

Genetic linkage analysis was performed using the software packages MSTmap [42] and JoinMap 4.0 [43]. The algorithm implemented in MSTMap can efficiently handle ultra-dense datasets from 10,000 to 100,000 markers, and independent comparisons of MSTMap with JoinMap have found it to produce the most accurate maps for most experiments with vey fast calculation times [44]. According to Wu et al. [42] the software generates extremely accurate map outputs when the data quality is high.

Polymorphic SNP markers were first grouped at LOD 5.0 using MSTmap, and then marker orders were determined by finding the minimum spanning tree of a graph for each linkage group based on pairwise recombination frequencies. The marker order and distance in each linkage group were recalculated and confirmed by Joinmap 4.0, applying the mapping function of Kosambi [45] and a minimum LOD score of 3.0. Marker pairs with zero recombination were assigned to the same genetic bin. A reference genetic map was constructed with SNP bins being designated according to the index number of the first SNP marker in each bin.

Detection of QTL for seed colour, cellulose, hemicellulose and ADL content were performed in the RIL population by composite interval mapping using the QTL Cartographer software version WinQTLCart2.5 [46]. The LOD threshold for detection of significant QTL was set by permutation analysis with 300 permutations. The linkage map and QTL positions were drawn using the software Mapchart [47].

## Results

### SNP map construction

**Figure 1:**
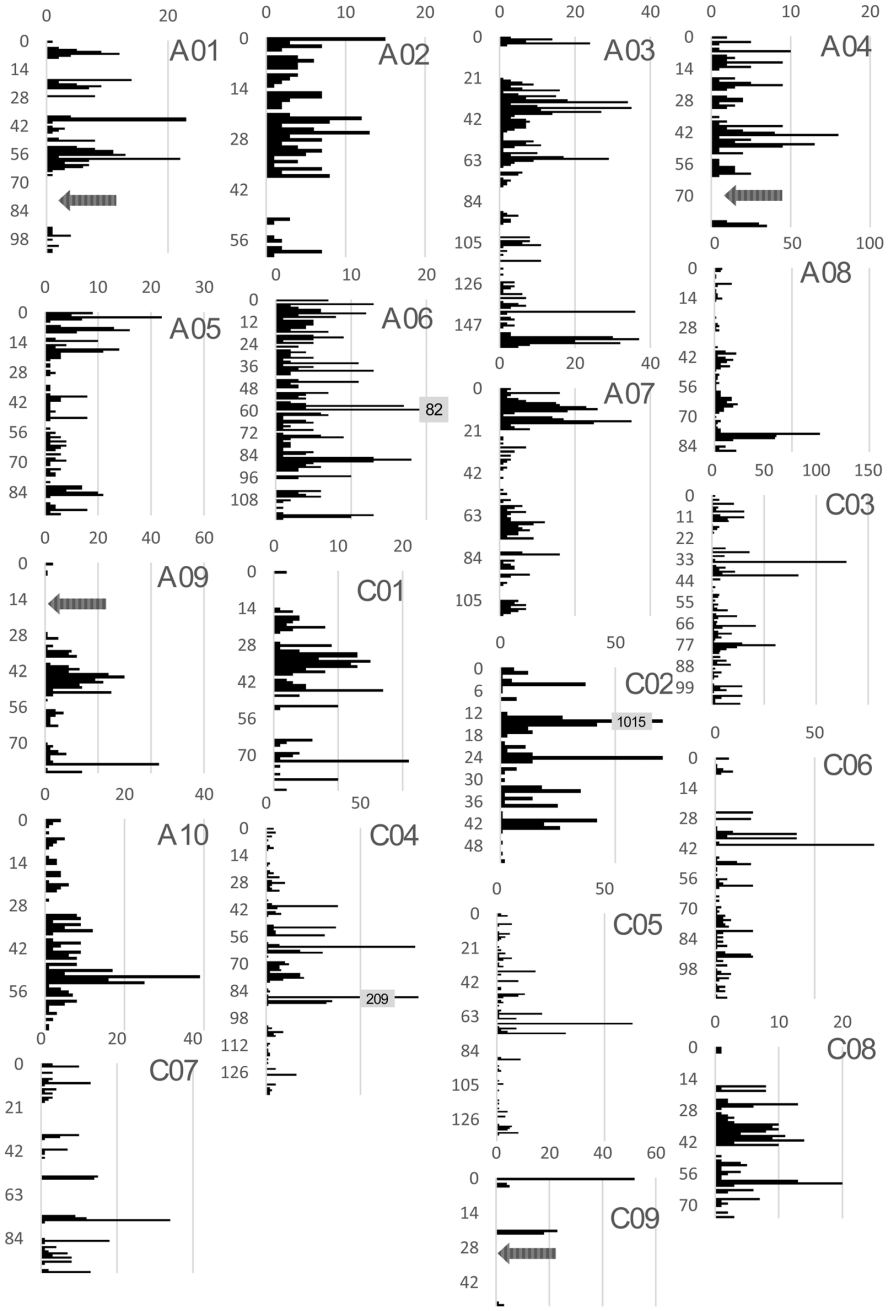
Overview of genome-wide SNP density in the *B. napus* SNP map. The abscissa indicates the number of SNP markers in each map interval, while the ordinate shows the genetic distance along each of the 19 linkage groups corresponding to the 19 *B. napus* chromosomes. The peak of the QTL was pinpointed to a narrow region at the terminal end of the chromosome flanked by two adjacent SNP markers.

A total of 16613 SNP markers from the Brassica 60 K array showed polymorphisms between the mapping parents GH06 and P174. Of these, 9804 homologous SNP markers showing the expected segregation 1:1 ratio in the RIL population were used for genetic linkage analysis AND linkage map construction using MSTmap and Joinmap 4.0. SNP markers expected to be specific to the *B. napus* A and C genome were cleanly separated into different linkage groups according to their chromosomal origin by using MSTmap at LOD 5, and some groups remained intact even at LOD 10. Finally, a set of 9164 SNP markers were successfully assigned to linkage groups representing the 19 *B. napus* chromosomes of the A genome (A01–A10) and C genome (C01–C09), respectively. As expected from the variable size of the *B. napus* chromosomes [48] and from previous genetic mapping studies, the marker number and density varied considerably across the different chromosomes (Figure 1). In the A genome three chromosomes displayed gaps of more than 20 cM, while 1 chromosome in the C genome also showed a gap over over 20 cM. The highest marker density was found on chromosome C02, with 1507 markers distributed over a genetic map distance of 52.4 cM.

A SNP bin map comprising 1232 bins was constructed (File S1). The number of SNP markers per bin varied from 2 to 950. The largest bin on C02 containing 950 markers, and only 504 markers from this bin gave successful BLAST hits in the *B.oleracea* database to chromosome C02.

The final high density *B. napus* linkage map for the GH06 x P174 RIL population contains a total of 2795 distinct loci, including 976 loci on C genome chromosomes and 1819 loci in the A genome. The map covers 1832.9 cM, with total chromosome lengths of 970.2 cM and 862.7 cM for the A and C genomes, respectively (File S1). The marker density in A genome was thus considerably higher than in the C genome, with an average distance between markers of 0.53 cM in the A genome and 0.93 cM in the C genome, respectively (Table 1).

**Table 1:** Summary of locus numbers, marker numbers, multiple-marker bins and linkage group genetic distances of the 19 A-genome (A01–A10) and C-genome (C1–C9) chromosomes in the *Brassica napus* SNP bin map.

Chromosome	Number of multi-marker bins	Number of markers in bins	Total number of markers	Total number of mapped loci (including bins)	Map length (cM)	Average distance between loci (cM)
A01	47	192	285	140	104.2	0.74
A02	37	123	201	116	60.2	0.52
A03	141	579	764	328	158.9	0.49
A04	40	124	191	106	83.9	0.79
A05	58	192	287	152	94.5	0.62
A06	93	392	548	249	119.0	0.48
A07	91	333	462	220	112.7	0.51
A08	81	350	435	166	86.7	0.52
A09	59	239	373	193	81.6	0.42
A10	62	214	301	149	68.5	0.46
C01	47	223	259	82	83.7	1.02
C02	62	1465	1507	103	52.4	0.51
C03	106	947	1033	191	107.7	0.56
C04	86	975	1032	142	137.6	0.97
C05	52	299	340	144	142.2	0.99
C06	80	481	543	142	111.5	0.79
C07	35	219	248	63	100.6	1.59
C08	46	176	222	92	75.1	0.82
C09	9	125	133	17	51.9	3.05
A genome	709	2738	3847	1819	970.2	0.53
C genome	523	4910	5317	976	862.7	0.88
Total (A+C)	1232	7648	9164	2795	1832.9	0.66

Markers showing identical genotype scores across the entire RIL population were grouped into a single bin and a single marker from each bin was used for QTL mapping. The number of mapped loci on each linkage group is the sum of the number of marker bins plus the number of individual markers not assigned to bins.

### QTL analysis using the high-density SNP map

**Figure 2:**
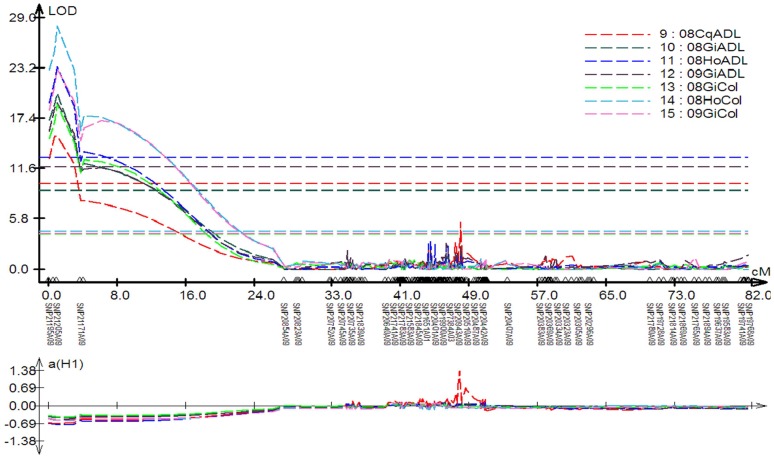
Graph showing a major QTL with large additive effects acid detergent lignin (ADL, four environments) and seed colour (Col, three environments) plotted according to logarithmic odds (LOD) score across the length of linkage group A09. Locations: Cq, Chongqing China; Ho, Hohenlieth Germany; Gi, Giessen Germany. Years: 08, 2008; 09, 2009.

**Figure 3:**
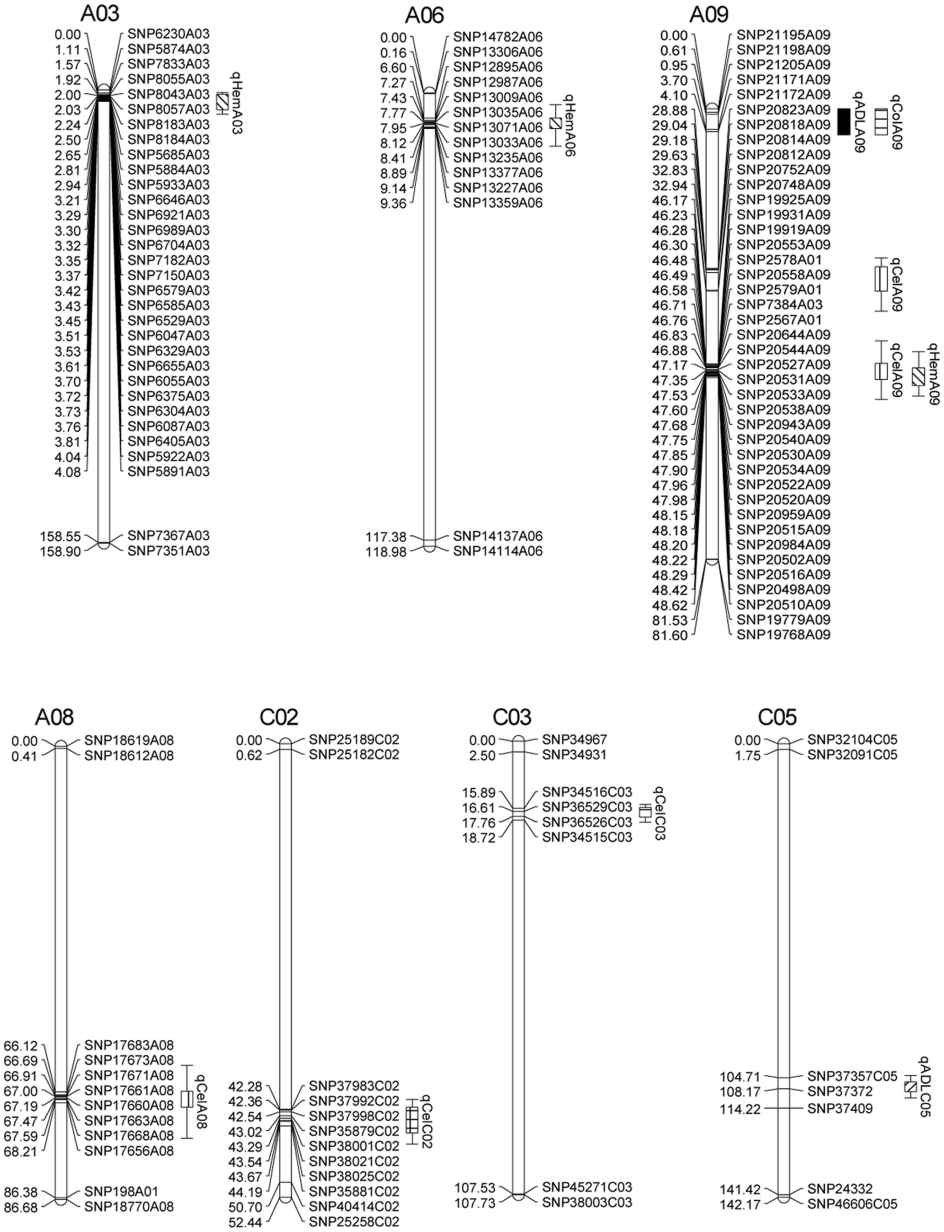
Locations of significant QTL for acid detergent lignin (ADL), cellulose (Cel), hemicellulose (Hem) and seed colour (Col) in four different environments on the high-density SNP map. For simplicity only the markers in the QTL confidence intervals, along with the terminal two markers at each end of the QTL-containing chromosomes, are shown. Full map data is provided in File S1.

A single major QTL explaining 36.9% to 47.2% of the phenotypic variation for seed colour across the three different environments was detected at the end of linkage group A09 (Table 2, Figure 2, Figure 3). Two QTL for seed ADL content were localised on chromosomes A09 and C05, accounting for 31.6–42.8% and 8.1–14.1% of the phenotypic variation in the four different environments, respectively (Table 2, Figure 2 and Figure 3). The negative additive effects of these three QTL indicated that the yellow-seeded parent GH06 contributed to a strong decrease in seed colour and seed ADL content. The QTL for seed colour and ADL on chromosome A09 showed perfect colocalisation- This is presumably indicative of a strong pleiotropic effect caused by reduced seed coat thickness [36], which also explains their high correlation of R = 0.67 in the RIL population (File S2). The confidence interval of these two QTL spans a physical region from 32.22 Mbp to 32.84 Mbp in the *B. rapa* A genome reference sequence (Figure 2). We scanned the 620 kbp physical interval between the SNP markers flanking the QTL confidence interval for potential candidate genes involved in seed phenylpropanoid (lignin biosynthesis) or proanthocyanin pigmentation (flavanoid biosynthesis). The flavonoid biosynthesis gene Bra007813, homologous to *FLAVONOL 3-HYDROXYLASE* (*F3H*, also known as *TRANSPARENT TESTA 6*, *TT6*) was located 32.52 Mbp on A09. In the flavonoid biosynthetic pathway F3H catalyzes naringenin to dihydrokaempferol, a key precursor of proanthocyanins that accumulate as dark seed pigments (condensed tannins) in the testa of *Brassica* seeds. Other lignin biosynthesis and flavonoid genes which previous studies have suggested as potential candidates for the major QTL for seed colour and fibre content on chromosome A09 [36], [38], [41], were shown to be outside the narrow QTL confidence interval calculated using the high-density SNP map.

**Table 2:** Significant QTL associated with seed colour and fiber traits in the GH06 x P174 F_9_ RIL population (n = 172) after phenotyping in 4 different field environments.

Trait	Environment	QTL	Chromosome	Marker interval	LOD score	Additive effect	R^2^ (%)
ADL content (% DW)	2008 Cq	***qADLA09***	A09	SNP21195A09-SNP21172A09	14.31	−0.66	32.8
		***qADLC05***	C05	SNP37372-SNP37409	4.52	−0.32	9.1
	2008 Gi	***qADLA09***	A09	SNP21195A09-SNP21172A09	15.39	−0.45	33.3
		***qADLC05***	C05	SNP37372-SNP37409	9.54	−0.28	14.1
	2008 Ho	***qADLA09***	A09	SNP21195A09-SNP21172A09	20.72	−0.75	42.8
		***qADLC05***	C05	SNP37372-SNP37409	5.33	−0.31	8.1
	2009 Gi	***qADLA09***	A09	SNP21195A09-SNP21172A09	15.2	−0.53	31.6
		***qADLC05***	C05	SNP37372-SNP37409	6.95	−0.28	13.1
Seed colour	2008 Gi	***qColA09***	A09	SNP21195A09-SNP21172A09	17.8	−0.44	36.9
	2008 Ho	***qColA09***	A09	SNP21195A09-SNP21172A09	23.6	−0.62	47.2
	2009 Gi	***qColA09***	A09	SNP21195A09-SNP21172A09	18.56	−0.53	39.1
Cellulose content (% DW)	2008 Cq	***qCelA08***	A08	SNP17683A08-SNP17656A08	6.51	−0.18	13.5
		*qCelA09*	A09	SNP20823A09-SNP20748A09	11.33	0.23	21.9
	2008 Gi	***qCelA08***	A08	SNP17683A08-SNP17656A08	6.27	−0.32	12.1
		*qCelA09*	A09	SNP19925A09-SNP20492A09	5.38	0.55	4.7
	2008 Ho	***qCelA08***	A08	SNP17683A08-SNP17656A08	8.86	−0.31	15.1
		*qCelC02*	C02	SNP37983C02-SNP35881C02	4.41	−0.20	6.4
		*qCelC03*	C03	SNP34516C03-SNP34515C03	5.85	−0.25	7.9
	2009 Gi	***qCelA08***	A08	SNP17683A08-SNP17656A08	6.91	−0.31	14.0
		*qCelC03*	C03	SNP34516C03-SNP34515C03	5.95	−0.25	7.5
Hemi-cellulose (% DW)	2008 Gi	***qHemA03***	A03	SNP7833A03-SNP5891A03	6.29	−0.22	8.3
	2008 Ho	***qHemA03***	A03	SNP7833A03-SNP5891A03	4.23	−0.15	7.3
	2009 Gi	***qHemA03***	A03	SNP7833A03-SNP5891A03	5.18	−0.18	8.3
		*qHemA06*	A06	SNP12895A06-SNP13359A06	5.51	0.19	10.2
		*qHemA09*	A09	SNP20527A09-SNP20457A09	4.68	−0.24	16.9

QTL were localized on high-density SNP map to allow navigation directly from QTL confidence intervals to genomic sequence regions of interest. Negative additive effect values indicate a decrease in the trait value caused by QTL alleles from parent GH06, while positive additive values indicate increasing trait values with QTL alleles from parent GH06. *R* ^2^ is the percentage of variation explained by each QTL. *Cq*: Chongqing, China; *Ho*, Hohenlieth, Germany; *Gi*: Giessen Germany. QTL consistent over different environments are shown in bold font.

Based on the high density linkage map, five QTL with phenotypic effects on seed cellulose content ranging from 4.7% to 21.9% were detected across the four different environments. For four of these QTL the alleles from GH06 contributed to a decrease in seed cellulose content, whereas the decrease in cellulose caused by QTL *qCelA09* on chromosome A09 was contributed by P174. The QTL with the greatest effect on cellulose content, *qCelA08*, was detected across all four environments on chromosome A08 with an effect of 12.1% to 15.1% on the phenotypic variation. A single QTL for hemicellulose content was localised on chromosome A03, with a phenotypic effect of around 8% on this trait in the three German environments.

## Discussion

The identification of genome-wide SNP markers for *B. napus* based on growing quantities of next-generation sequencing data has opened the possibility for development of cost-effective, high-density SNP screening arrays for high-throughput genotyping of large oilseed rape mapping and breeding populations [12]. Whereas most QTL studies in *B. napus* in the past were based on lower-density genetic maps, often without sequence annotation of the marker loci, high-density SNP maps open the possibility for accurate alignment of *B. napus* genetic maps to physical chromosome segments in assembled *Brassica* genomes. This greatly increases the utility of genetic linkage maps for characterisation and comparison of QTL controlling important agronomic traits.

The marker density of a genetic map cannot be increased solely by increasing the number of markers used for genotyping, since physically linked markers will co-segregate on chromosome segments that show no recombination in a given population. By use of a F9 RILs mapping population we were able to capture a large amount of recombination in our mapping study, greatly increasing the number of individual loci that were able to be mapped to discrete genetic map positions. On this regard, one great advantage of robust SNP array screening platforms like the Brassica 60 k SNP BeadArray is the low proportion of missing data. Other high-density marker types, like Diversity Array Technology (DArT) or sequencing-based restriction site-associated DNA (RAD) markers, can be problematic for accurate genetic mapping because of the large amount of missing data normally associated with such methods: Missing data can be very difficult to distinguish from real polymorphisms and can therefore introduce significant errors into a high-density genetic map. To reduce mapping errors and avoid artificial exaggeration of map distances, missing data form must be dealt with using complex imputation analyses [49]. Furthermore, the use of high-throughput screening arrays enables automated assaying of very large numbers of markers in a very short time (in our case 52157 SNP loci in 174 genotypes in around 3 days).

At 1832.4 cM the length of the SNP linkage map was somewhat longer than a previous 1589 cM map from a younger generation of the same population which was constructed using SSR, SRAP and AFLP markers [50]. This minor increase in map length is expected due to the huge increase in the number of mapped loci. The length is similar to that of the map reported by Chen et al. [16] for 8827 sequence-derived SNP loci mapped over 1860 cM. About 80 cM of our map was accounted for by 4 gaps of around 20 cM. These may result form of distorted segregation caused by rearrangements among homoeologous chromosomes in one or both of the mapping parents [51]. Interestingly, the marker density and recombination frequency was considerably higher in in A genome than the C genome in this population. This may reflect a frequent implementation of diverse *B. rapa* (A genome) germplasm in breeding of Chinese rapeseed, which may have reduced the extent of linkage disequilibrium and higher polymorphism in the A genome of the mapping parents used in this study. The largest bin, containing 950 SNP markers on chromosome C02, provides an example for a particularly recombination-poor region of the C genome in this cross. Interestingly, a relatively large number of SNP markers from the C genome of *B. napus* gave no BLAST hit in the *B. oleracea* database. Such results suggest signficant genome-scale differences between *B. napus* and its C-genome progenitor *B. oleracea*.

Almost two decades after the first genetic mapping of seed colour loci in *B. napus* [22] the responsible genes are still unclear. The considerably higher accuracy for QTL detection enabled by a higher map density [13] now gives us the opportunity to more accurately localise candidate genes and compare different mapping studies via the genome sequence. In a previous study we fine-mapped the major locus for seed colour and ADL to one side of SSR marker KBrH092O19.5_400, however no polymorphic reference markers could be identified in the nearby chromosome sequence on the other side of the QTL. In the present study the SNP marker SNP21195A09 was identified to the other side of the QTL peak, enabling the QTL to be clearly located on the *B. rapa* genome sequence. Xiao et al. [39] mapped a recessive gene for seed colour on chromosome A09 in *B. rapa*, however the physical region containing this gene (between 19.3 and 22.1 Mbp) does not correspond to the locus identified in our study. Furthermore, Kebede et al [40] mapped a major seed colour QTL in *B. rapa* on the middle of A09. These contrasting results suggest that different genes on chromosome A09 are involved in seed colour variation in different genetic backgrounds in *B. rapa* and *B. napus*. A major locus influencing seed colour and ADL content in a genetically diverse winter oilseed rape background was also mapped by Stein et al. [41] on A09. The most closely and consistently linked marker in that study, Ni4D09, is located at 32.89 Mbp in the *B. rapa* sequence, only 4 kpb from the plasma membrane proton-ATPase Bra039228. This gene is an orthologue of another Arabidopsis *TRANSPARENT TESTA* gene, *AHA10*, which is a seed-expressed H+-ATPase pathway, Bra007813, was found within the QTL confidence interval, Bra007813 is a homolog of *FLAVANONE 3-HYDROXYLASE* (*F3H*, also known as *TRANSPARENT TESTA 6*) and thus another highly interesting candidate for seed variation. These results indicate that independent mutations in different phenylpropanoid genes may cause similar seed coat phenotypes in different genetic backgrounds.

To our knowledge this study is the first to report QTL for seed cellulose and hemicellulose content in *B. napus*. The environmentally consistent QTL on A08, which can reduce the seed cellulose content more than 10%, may be an interesting target for breeding to improve oil content. Both cellulose and hemicellulose showed negative correlations with seed oil content in the GH06 × P174 RIL population, demonstrating that reduction of cellulose and hemicellulose can improve the seed oil content due to a redirection of photosynthetic assimilates from sugar biosynthesis into seed oil biosynthesis. The two QTL qCelA09 and qHemA09 were found overlapped on A09 with opposite additive effects for cellulose and hemicellulose content, respectively. These two QTL may represent the same QTL with negative pleiotropic effects. Accurate mapping of this QTL represents a first step towards marker development and gene identification for potential improvement of oil content.

## Supporting Information

File S1 **Map data with bins.** Full list of SNP markers and bin map positions for the 19 *B. napus* chromosomes along with a summary of map distances and bin numbers.
(XLSX)

File S2 **Fibre trait correlations.** Scatter plots and correlation coefficients among fiber traits across the different environments.
(XLSX)
